# Comparative evaluation of chromogenic agar medium and conventional culture system for isolation and presumptive identification of uropathogens

**DOI:** 10.12669/pjms.305.5243

**Published:** 2014

**Authors:** Laila Akter, Rezwana Haque, Md. Abdus Salam

**Affiliations:** 1 Laila Akter, M.Phil (Microbiol), Associate Professor, Department of Microbiology, Islami Bank Medical College, Rajshahi, Bangladesh.; 2 Rezwana Haque, M.Phil (Microbiol), Assistant Professor, Islami Bank Medical College, Rajshahi, Bangladesh.; 3 Md. Abdus Salam, PhD, FRCP, Associate Professor, Department of Microbiology, Rajshahi Medical College, Rajshahi-6000, Bangladesh.

**Keywords:** Urine culture, Chromogenic agar medium, Conventional culture system, Rate of isolation, Presumptive identification

## Abstract

***Objective:*** Urine is the most frequent specimen received for culture/sensitivity by clinical laboratories. The microbiological performance of HiCrome UTI agar medium was compared with Blood agar and MacConkey agar for isolation and presumptive identification of bacteria from urine culture.

***Methods:*** A total of 443 consecutively collected midstream and/or catheter-catch urine samples from patients attending the Islami Bank Medical College Hospital, Rajshahi, Bangladesh during January to December, 2012 were cultured. Urine samples showing pus cells ≥ 5/HPF were inoculated on to Blood agar (BA), MacConkey agar (MAC) and HiCrome UTI agar (CA) media simultaneously and incubated overnight aerobically at 37^0^C. Rate of isolation and presumptive identification of bacterial species were compared for different media.

***Results:*** Culture yielded a total of 199 bacterial isolates from 189 (42.67%) positive plates including 179 (40.40%) unimicrobial and 10 (2.26%) polymicrobial (mixed growth of pair of bacteria) growths. Both HiCrome UTI agar and Blood agar media supported 100% growths while 151 (75.88%) growths were observed on MacConkey agar. The rate of presumptive identification was found significantly higher on HiCrome UTI agar (97.49%) than MAC agar (67.34%) (P<0.001) as primary urine culture medium. Of 199 isolates, *E. coli *was found to be the leading uropathogen isolated from 118 (59.30%) samples with its presumptive identification rate of 95.76%, 93.22% and 5.93% on CA, MAC and BA respectively. All 10 (100%) polymicrobial growths were demonstrated distinctly on CA against only 01(10%) on each BA and MAC.

***Conclusion:*** HiCrome UTI agar was found to be more useful as primary urine culture medium in both higher rate of isolation and presumptive identification of uropathogens in comparison to conventional media. Its inherent characteristics in demonstrating polymicrobial growth and ease of rapid identification by distinct colony colour are unique.

## INTRODUCTION

Urinary tract infections are important clinical entities that account for significant outpatients load and hospital admissions globally, making urine, the most frequent sample received for culture.^1^ Although many of these infections are treated empirically, urine cultures involve a significant portion of clinical microbiology laboratory's daily workload.^2^^,^^3^ The etiological diagnosis of UTI requires quantitative urine culture on standard agar media, because only 20 to 30% of urine samples results in significant growth with predominant causative agents which are *E. coli,* *Klebsiella *spp.,* Pseudomonas *spp.,* Proteus *spp.* Enterococci *spp*.*, and *Staph. *saprophyticus.^4^^,^^5^

For urine culture, the media should ideally be able to support the growth of all urinary pathogens and inhibit possible contaminants. Traditionally conventional media like Blood agar (BA) and MacConkey agar (MAC) are being used in combination by most of the laboratories especially in the developing countries for long and Cystine lactose electrolyte-deficient (CLED) agar has been added later on but none of these media singly or in combination can support the growth and/or identification of possible uropathogens.^6^ As a result there is continuous strive by the laboratories to streamline and improve urine culture algorithms. Blood agar can support the growth of majority of uropathogens as an enriched medium but its performance in identification of bacteria is very poor. Similarly differentiation of lactose fermenter and non-fermenter is possible on MAC and CLED agar, but further species identification necessitates subculture or different biochemical tests with consequent longer reporting time and cost. Moreover, their limited capacities in maximizing the growth of possible pathogens rendered them unsuitable as an ideal primary isolation medium.^3^

The problem of urine culture has been addressed by the introduction of chromogenic agar (CA) medium which is commercially available for two decades. It is being increasingly used as a versatile primary culture tool for better isolation, presumptive identification and differentiation of bacterial species from clinical specimens.^7^ Chromogenic substrates are incorporated into these media that are broken down by bacterial enzymes imparting a distinct visible colour to the growing bacterial colonies for their identification.^8^ This single medium supports not only the growth of all uropathogens but mixed infections can also be diagnosed more easily.^9^ In a few studies comparing chromogenic media with traditional ones its advantages including 20% reduction in time for identification, reduction in workload^10^, easier recognition of mixed growth^9^^,^^11^ and reduction in number of biochemical tests for bacterial identification have been shown. All these factors have direct impact on ultimate cost reduction.

HiCrome UTI agar is such a chromogenic medium that facilitates rapid isolation as well as presumptive identification of most uropathogens including various species from mixed cultures.^12^ Colonies of *E. coli*, the most common uropathogen appear as pink-red because of β-galactosidase production thus allows its definite identification without need for further biochemical tests. Strains that produce β-glucosidase, such as *Enterococci* and the *Klebsiella-Enterobacter-Serratia* group form blue colonies result from hydrolysis of glucoside, a chromogenic substrate incorporated in the medium. Similarly, tryptophan is also present in the medium to detect members of the *Proteus *group, which generates a diffuse brown coloration as a result of tryptophan deaminase production.^13^ *Pseudomonas* spp. produces colourless colonies whereas *Staph. saprophyticus* produces white colonies.^14^ Certain identification tests like the catalase, oxidase and indole production can be done directly from the colonies on HiCrome UTI agar and antibiotic sensitivity testing without subculturing onto another basic medium is also possible.^15^

The present study was undertaken to validate the usefulness of HiCrome UTI agar as a primary urine culture medium for its rate of isolation and presumptive identification of uropathogens in comparison to BA and MAC in a teaching hospital in Bangladesh.

## METHODS

***Ethical concern: ***The protocol was approved by the Ethical Review Committee of Islami Bank Medical College, Rajshahi, Bangladesh.

***Sample collection: ***This retrospective cross-sectional study included 443 consecutively collected midstream and/or catheter-catch urine samples from clinically suspected UTI patients of different age and sex groups attended either at the outpatient department or admitted in the Islami Bank Medical College Hospital, Rajshahi, Bangladesh from January to December, 2012. Urine culture was performed for samples that showed pus cells ≥ 5/ HPF on microscopy of a centrifuged deposit of urine^5^. Patients were advised to collect clean-catch mid stream or catheter-catch urine into a sterile wide mouth container/test tube with all aseptic measures (information on how to collect proper sample in sterile container aseptically was given prior to collection).

***Preparation of media: ***HiChrome UTI agar, MacConkey agar and base for Blood agar media were obtained as a dehydrated powder from HiMedia laboratories (HiMedia Laboratories Pvt. Ltd. Mumbai-400086, India). All culture petri plates were prepared in house by following manufacturer’s instructions and recommendations. For preparation of Blood agar, 5% defibrinated sheep blood was used. Prepared plates were stored at 2-8^0^C for a month. Every fresh batch of media was tested for its ability to support the growth of *Escherechia coli* ATCC (25922) to ensure the quality of the media.

***Culture of urine: ***All urine samples were inoculated aseptically on to HiChrome UTI agar, Blood agar and MacConkey agar media by using a calibrated wire loop of 28G with an internal diameter of 3.26 mm holding 0.004 ml of urine. The plates were incubated at 37^0^C aerobically and after overnight incubation they were checked for significant bacteriuria as under by enumeration of colonies [growth of 100 colonies equals to 10^5^ colony forming units (cfu) of bacteria /ml of urine].^15^

***Criteria for significant bacteriuria: ***Presence of >10^5^ cfu/ml of non-coliforms or >10^2^ cfu/ml of coliforms in a symptomatic woman.

- Presence of >10^3^ cfu/ml of bacteria in a symptomatic man.
- Growth of two different organisms from possible uropathogens at a concentration of

10^4^ cfu/ml.

***Presumptive identification: ***Presumptive identification of bacterial growth was done on HiCrome UTI agar according to colony morphology and colour as depicted by the manufacturer. Colonies on the MAC agar and BA were also identified following colony characteristics against each of the uropathogens. The final identification of the isolates was done using standard identification protocol such as Gram’s staining, motility test, catalase test, coagulase test, oxidase test and other relevant biochemical tests as appropriate for the isolates.^15^

***Statistical Analysis: ***All data were entered into Statistical Package for Social Sciences (SPSS) version 16.0. Frequencies with percentage were generated for categorical variables such as, type of bacteria, rate of isolation, presumptive identification, and rate of polymicrobial growth in culture. P value was calculated using Chi-square (*x*^2^) test for comparison of presumptive identification rate of CA and MAC.

## RESULTS

Out of 443 urine culture, 189 (42.67%) yielded significant bacterial growths and 254 (57.33%) showed no growth. Culture-positive samples included 179 (40.41%) growth of single organism and 10 (2.26%) mixed growth of two organisms each.

Patterns of bacterial isolates from urine culture are shown in Table-I. 189 culture-positive samples yielded a total of 199 bacterial isolates including 179 single and 10 (two bacteria in each plate account for 10x2=20 isolates) polymicrobial growths. *E. coli* was the leading bacteria isolated from 118 (59.30%) samples followed by* Staph. saprophyticus* 38 (19.09%),* Enterococcus *spp. 23 (11.56%), *Klebseilla *spp. 11 (5.53%), *Pseudomonas *spp. 04 (2.01%),* Proteus *spp. 03 (1.51%) and* Enterobacter s*pp. 02 (1.00%).

Comparative results of three culture media for their rate of isolation of uropathogens are shown in Table-II. It was observed that both HiCrome UTI agar and blood agar media supported 100% bacterial growth, while 151 (75.88%) growths were observed in MAC agar.

For presumptive identification of bacterial species by colony characteristics on primary culture plate, of 199 bacterial isolates, 194 (97.49%) could be differentially identified on HiCrome UTI agar against 134 (67.34%) and 73 (36.68%) on MAC agar and BA respectively. The rate of presumptive identification of the isolates was found significantly higher (P<0.001) on HiCrome UTI agar than MAC agar as primary urine culture medium (Table-III)**.**

Table-IV shows the rate of presumptive identification of polymicrobial growth in different culture media. All 10 (100%) polymicrobial growths were distinctly identified only on HiCrome UTI agar medium, while except in a single case consisting of *E. coli* and *Proteus *spp., all other mixed bacterial growths could not be identified on both MAC and Blood agar media.

## DISCUSSION

Presumptive identification of bacterial isolates in urine culture is time consuming and requires a great deal of experience when using traditional media like Blood agar, MacConkey agar or CLED agar. On the contrary, HiCrome UTI agar medium was found to be much superior over conventional media for its higher rate of isolation and uniform interpretation for identification of uropathogens. As many of the extra tests for bacterial identification associated with conventional culture methods were no longer required, chromomeric medium substantially reduced the laboratory workload with concomitant high bench throughput.

**Table-I:** Pattern of bacteria isolated from urine culture (N=199).

***Bacteria***	***Number (%)***
*E. coli*	118 (59.30)
*Staph. saprophyticus*	38 (19.09)
*Enterococcus *spp.	23 (11.56)
*Klebsiella *spp.	11 (5.53)
*Pseudomonas *spp.	04 (2.01)
*Proteus *spp.	03 (1.51)
*Enterobacter *spp.	02 (1.00)
**Total**	**199 (100)**

**Table-II:** Comparison of three culture media for the rate of isolation of uropathogens

***Bacteria (Number)***	***HiCrome UTI agar N (%)***	***MAC agar N (%)***	***Blood agar N (%)***
*E. coli *(118)	118 (100)	118 (100)	118 (100)
*Staph. saprophyticus *(38)	38 (100)	00	38 (100)
*Enterococcus *spp. (23)	23 (100)	13 (56.52)	23 (100)
*Klebsiella *spp. (11)	11 (100)	11 (100)	11 (100)
*Pseudomonas *spp. (04)	04 (100)	04 (100)	04 (100)
*Proteus *spp. (03)	03 (100)	03 (100)	03 (100)
*Enterobacter *spp. (02)	02 (100)	02 (100)	02 (100)
**Total (199)**	**199 (100)**	**151 (75.88)**	**199 (100)**

**Table-III:** Comparison of media for rate of presumptive identification as primary culture plate

***Bacterial strains (Number)***	***HiCrome UTI agar N (%)***	***MAC agar N (%)***	***Blood agar N (%)***
*E. coli *(n=118)	113 (95.76)	110 (93.22)	07 (5.93)
*Staph. saprophyticus *(n=38)	38 (100)	00	38 (100)
*Enterococcus *spp. (n=23)	23 (100)	08 (34.78)	15 (65.22)
*Klebsiella *spp. (n=11)	11 (100)	09 (81.82)	08 (72.73)
*Pseudomonas *spp. (n=4)	04 (100)	03(75.00)	02 (50)
*Proteus *spp. (n=3)	03 (100)	03 (100)	03 (100)
*Enterobacter *spp. (n=2)	02 (100)	01 (50)	00
**Total (199)**	**194 (97.49)**	**134 (67.34)**	**73 (36.68)**

**Table-IV:** Comparison of rate of isolation of polymicrobial growth on culture media (N=10).

***Organism pair***	***HiCrome UTI agar N (%)***	***Blood agar N (%)***	***MAC agar N (%)***
*E. coli & Staph. saprophyticus*	04 (40)	Nil	Nil
*E. coli & Enterococci* spp.	03 (30)	Nil	Nil
*E. coli *& *Klebsiella* spp.	02 (20)	Nil	Nil
*E. coli* & *Proteus* spp.	01 (10)	01 (10)	01 (10)
**Total**	**10 (100)**	**01 (10)**	**01 (10)**

The rate of isolation and pattern of major uropathogens of the present study are in accordance with a few studies carried out on both chromogenic and conventional media.^3^^,^^12^^,^^16^^,^^17^^,^^18^ It has generally been noted that only 20 to 30% of urine samples results in significant growth with predominant causative agent being *E. coli* in both community and hospital acquired infections .^4^^,^^8^^,^^16^^,^^17^ Regarding rate of uni and polymicrobial growths from urine culture, our results corroborate with a few studies done here and in India.^16^^,^^17^^,^^18^ In contrast a higher rate of isolation was reported from a study in UK (54.2% single growth and 21.6% mixed growth).^19^ This difference might be due to inclusion of urine samples for culture having pus cell > 200/cmm in that study.

HiCrome UTI agar and Blood agar supported the growths of all 199 (100%) isolates whereas MacConkey agar yielded 151(75.88%) bacterial growths. Blood agar is an enriched medium and HiCrome UTI agar also contains all essential nutrients to support the growth of possible uropathogens that is why all isolates were possible to be grown on to these two media and similar findings were also reported by others.^14^^,^^16^ Slightly lower yielding rate on MAC agar can be explained by its limitations of not supporting all organisms involved in UTI like *Staph. saprophyticus* and *Enterococcus *spp., because it is a selective medium for members of Enterobacteriaceae.

As far as the presumptive identification of bacterial species is concerned, significantly high percentage of bacterial species were possible to be identified on HiCrome UTI agar by matching with standard colours as opposed to conventional culture system. This high rate of identification could be correlated with the ease of identification technique by seeing the distinct and perceivable colony colour produced by each of the bacterial species on chromogenic agar medium and our findings are consistent with reports published elsewhere.^9^^,^^12^^,^^16^ There was significant difference in rate of presumptive identification especially for *E. coli*, *Klebsiella* spp., *Enterococci* spp. and *Enterobacter* spp. on HiCrome UTI agar and similar results were also observed by other investigators.^16^^-^18 In fact, this differential colour production by individual bacterial species is among the most exciting features of chromogenic agar for which it has been advocated to be used as primary urine culture medium. The chromogenic media also provided added advantage on identification of a few non-lactose variety of *E. coli*, which might be the reason of decreased rate of identification on MAC agar. Moreover, HiCrome UTI agar offered the advantage of limiting the spread of some isolates such as *Proteus* spp., *Klebsiella *spp. and *E. coli* mucoid strains thus increased the ability of the medium to detect urinary tract pathogens when mixed organisms were present.^7^^,^^8^

The HiCrome UTI agar also reigned over the conventional media by providing high isolation rate as well as specific identifying characteristics of the organisms in mixed growth thus enabling microbiologists to assess more accurately the clinical relevance of urine culture results. Similar findings for polymicrobial growth in chromogenic agar were also reported by a few investigators.^16^^-^^19^ The rate of identification of mixed culture on the BA and MAC agar was very poor due to their limitations in differentiating the colonies. Improved detection of mixed cultures may help to identify contaminated specimens and therefore lead to a reduction in the prescription of unnecessary antibiotics.^19^ Now it is obvious from the results of the present and similar studies that chromogenic medium has the right potential to replace both CLED and MAC agar as primary urine culture medium because of its superiority in rate of isolation and ease in identification through characteristic colony colour. Moreover, it also provides an added advantage of requiring less time in mastering the skill in identification of the uropathogens in contrast to conventional media. Though chromogenic media on its own are still expensive at the moment but considering the overall costs incurred for the use of multiple media and/or different biochemical tests necessary to identify the organism in the conventional urine culture system, it is cost-effective indeed.

## CONCLUSION

The overall findings of this study suggests and reinforced that HiCrome UTI agar offers an excellent and time saving method for the reliable identification of most of the uropathogens and differentiation of mixed bacterial cultures in primary culture plate as opposed to conventional culture system. It has the potential to streamline urine culture processing in a meaningful way, such as reducing technologist workload, improving result turnaround times and reducing costs which together all have considerable laboratory impact.

## Author’s contributions:

**MLA and RH** have conceived the study, performed the laboratory works and drafted the manuscript. **MAS** has contributed for intellectual thoughts, study design, statistical analysis and critical editing of the manuscript. All authors have read and approved the submitted version of the manuscript.
